# Medication Responsiveness of Motor Symptoms in a Population-Based Study of Parkinson Disease

**DOI:** 10.4061/2011/967839

**Published:** 2011-12-08

**Authors:** Yvette M. Bordelon, Ron D. Hays, Stefanie D. Vassar, Natalie Diaz, Jeff Bronstein, Barbara G. Vickrey

**Affiliations:** ^1^Department of Neurology, David Geffen School of Medicine at UCLA, Los Angeles, CA 90095-7334, USA; ^2^Department of Medicine, UCLA Division of General Internal Medicine and Health Services Research, Los Angeles, CA 90095-1736, USA; ^3^UCLA School of Public Health, Los Angeles, CA 90095-1772, USA; ^4^RAND, Santa Monica, CA 90407-2138, USA; ^5^Parkinson's Disease Research, VA Greater Los Angeles Healthcare System, Education and Clinical Center, Los Angeles, CA 90073, USA; ^6^Department of Neurology, Harbor-UCLA Medical Center, Torrance, CA 90502, USA

## Abstract

We assessed degree of Parkinson disease motor symptom improvement with medication among subjects enrolled in an ongoing, population-based study in Central California. The motor section of the unified Parkinson disease rating scale (UPDRS) was performed on subjects in both OFF and ON medication states, and difference between these scores was used as an indicator of symptomatic benefit. Higher OFF minus ON scores correlated with more severe baseline symptoms. There was equivalent improvement on the motor UPDRS scale for subjects divided according to medication classes used: levodopa alone 7.3 points, levodopa plus other medications 8.5 points, and dopamine agonists but not levodopa 6.1 points. In addition, there was no difference in the magnitude of improvement when subjects were divided according to Parkinson disease subtype, defined as tremor dominant, akinetic-rigid, or mixed. In this community-based sample, these values are within the range of a clinically important difference as defined by previous studies.

## 1. Introduction

Clinical trials in Parkinson disease document improvement of motor features of the disorder using standardized rating scales, in particular the unified Parkinson disease rating scale (UPDRS). However, subjects participating in these studies generally seek care in tertiary care centers; due to strict procedures implicit in the conduct of clinical trials, such subjects' medications and response to medications are optimized and tightly controlled. To assess PD motor symptom control in a community-dwelling population, we used a cohort of PD subjects followed in a population-based study of PD risk and predictors of progression [1]. These subjects reside in rural counties in Central California, and their PD care is managed by local general neurologists [1]. Subjects were examined by movement disorder specialists using UPDRS Motor exams in the OFF state and again after their PD medication dosage (ON exam) as prescribed by their community clinician. The difference in Motor UPDRS scores between the OFF and ON states was used to document the degree of PD symptom improvement. It has been previously established by Shulman and colleagues that Motor UPDRS OFF-ON differences are clinically important with ranges of 2.3–2.7 points for minimal CID (clinically important difference) and 10.7–10.8 for large CID [2]. We investigated whether the Motor UPDRS OFF-ON differences found in our study were within these ranges and if the degree of improvement was associated with any factors in particular. Comparisons were made according to medication class used as levodopa has more pronounced symptomatic benefit than dopamine agonists and might result in greater OFF-ON differences [3, 4]. We also assessed whether PD subtypes may respond differentially to medications, knowing that akinetic-rigid PD typically exhibits a more rapid decline than tremor-dominant PD and is less responsive to treatments [5].

## 2. Methods

Subjects with idiopathic Parkinson disease (PD) identified within the prior three years were enrolled in the Parkinson's environment and gene (PEG) study on risk factors of incident PD [1]. General neurologists practicing in and around Kern, Tulare, and Fresno counties referred PD patients to the PEG study, and among referrals, approximately 45% fell within the three-year diagnosis duration required for study entry. Overall, 28 of the 31 neurologists in these counties (90%) referred patients. Study subjects completed questionnaires, and their diagnosis was confirmed by a movement disorders neurologist. Study participants returned to receive routine care for their PD by their local general neurologists. Enrolled subjects were then invited to participate in a follow-up study. Of the 371 subjects initially enrolled into PEG, 254 were eligible and provided written, informed consent for the current study (UCLA Institutional Review Board no. G06-07-055), and were reevaluated as part of the follow-up study between June, 2007 and June, 2009. Subjects provided updated information, including medications, and underwent two motor examinations by one of four board-certified neurologists with movement disorders fellowship training experienced in UPDRS administration. Examiners were unaware of this study's planned analyses at the time of data collection.

The UPDRS Motor examination section and Hoehn and Yahr staging were performed for each subject in both the practically defined OFF medication state (patients willing and able to withhold all PD medications for at least 12 hours prior to assessment) and the ON medication state (one hour after taking their usual morning PD medications that same day). The motor section contains 27 items scored 0 (no impairment) to 4 (severe impairment) with a possible scale score range of 0–108 [6]. Difference in OFF and ON medication state Motor UPDRS scores was examined by ON score and by OFF score using scatterplots.

Subjects were divided into three subgroups by medication used: levodopa only, levodopa plus other PD medication (including monoamine oxidase-B inhibitors, dopamine agonists, catechol-o-methyl transferase inhibitors, amantadine, or trihexyphenidyl), and dopamine agonists without levodopa plus other PD medication. The difference between OFF and ON Motor UPDRS exam scores was calculated for each subject. One-way analysis of variance (ANOVA) among medication subgroups by difference between OFF and ON state Motor UPDRS exam scores was determined. Chi square analysis of Hoehn and Yahr stage was performed among medication subgroups and PD subtypes.

PD subtypes were defined on the basis of the OFF state Motor UPDRS exam according to criteria used for subtype analysis of the initial PEG population, with minor modification [1]. Tremor-dominant PD was defined as a ratio >1.0 of tremor score (sums of items 20 and 21 divided by 7) divided by the akinetic/rigid score (sum of items 22–31 divided by 18). Akinetic-rigid PD was assigned when this ratio was <0.8, and mixed subtype to subjects with a ratio between 0.8 and 1.0.

## 3. Results

Of 254 study participants, 193 completed both OFF and ON PD medication assessments and were included in these analyses. Of those excluded, 24 could not undergo an in-person evaluation, 16 could not withhold morning medications (only ON exam performed), and 21 completed only the OFF evaluation, as they were not on PD medications (*n* = 12), did not bring their medications (*n* = 3) or no reason was recorded (*n* = 6). Average age was 72.4 years (SD 9.2), and there was a male (59%) and white (79%) predominance as in the initial cohort [1]. Mean disease duration was 5.2 years (SD 2.3), and 82% of subjects were Hoehn and Yahr stage 2 or higher (Table 1). Breakdown of medications used and mean doses are provided in Table 2.

**Figure 1:**
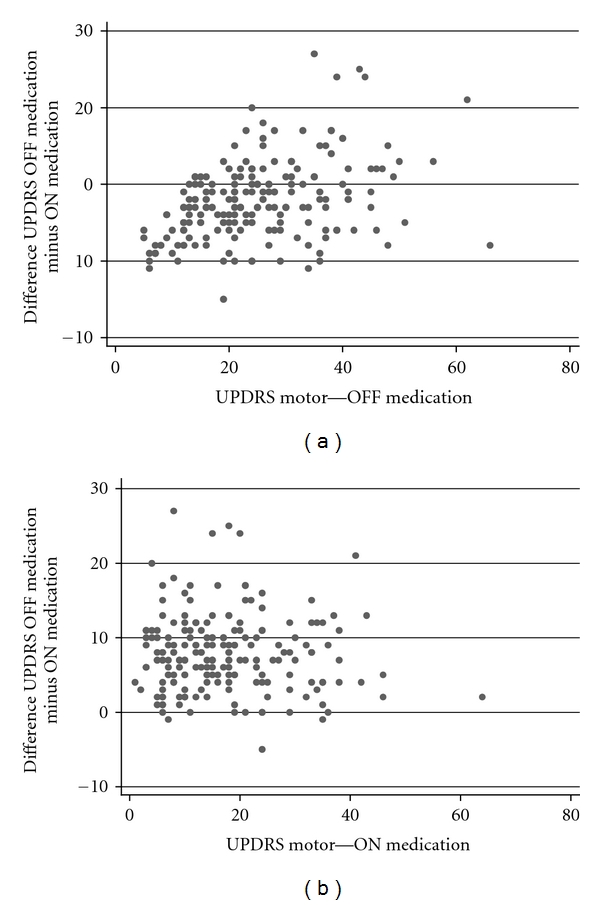
(a) Scatterplot of total Motor UPDRS scores OFF PD medication versus difference in total Motor UPDRS scores OFF medication and ON medication (test of slope *P* = 0.01). (b) Scatterplot of total Motor UPDRS scores ON PD medication versus difference in total Motor UPDRS scores OFF medication and ON medication (test of slope *P* = 0.46).

Scatterplots of the difference between OFF and ON Motor UPDRS score against the OFF state (Figure 1(a)) and ON state (Figure 1(b)) score revealed that higher OFF Motor UPDRS scores correlated with greater medication responsiveness (bigger improvement in Motor UPDRS scores from OFF to the ON state) (test of slope *P* = 0.01). Thus, there was clear improvement in motor symptoms with PD medications, more pronounced in more advanced disease. However, when medication response was plotted against ON scores there was no significant correlation (test of slope *P* = 0.46), indicating that most subjects achieved similar levels of symptom control (equivalent ON scores) regardless of underlying disease severity.

We next explored whether certain features determined the degree of medication responsiveness in this community-dwelling cohort of PD patients. Subjects were divided into three groups according to PD medications used. One subject was not categorized in this scheme, as they were on MAO-B inhibitors alone. The majority of subjects (91%) were on levodopa either alone or in combination with other PD medications. Comparisons were made among medication subgroups and ON, OFF, and difference between OFF and ON Motor UPDRS scores (Table 3). Baseline OFF medication scores were lower (better) in subjects not on levodopa (*P* < 0.05 pairwise *t*-test), which is to be expected, as in general, levodopa may be added later as disease progresses. However, medication response as measured both by ON scores and by OFF-ON scores was not different across the medication subgroups, with improvement in Motor UPDRS scores ranging from 6.1–8.5 points. There was no difference among medication subgroups in Hoehn and Yahr staging (Table 3 Chi square).

In the analysis of OFF-ON Motor UPDRS score differences by PD subtype, 33 subjects were classified as tremor-dominant, 142 as akinetic-rigid and 18 as mixed. The akinetic-rigid subtype had higher Motor UPDRS scores in the OFF and ON states than tremor-dominant or mixed subgroups indicating greater disease severity (Table 4). Mean daily levodopa dosage was not different among the subtypes (Table 4). However, the difference in OFF and ON scores was the same across all subtypes, demonstrating that regardless of motor symptom severity, PD medications led to similar range of improvement of 7 to 8 UPDRS points. There was a tendency for akinetic-rigid subjects to have a higher Hoehn and Yahr stage indicative of more severe disease (Table 4 Chi square), yet they responded to a similar degree as the other subtypes. A subgroup analysis of only those subjects with Hoehn and Yahr ≤2.5 demonstrated that the OFF-ON score differences for tremor-dominant subjects was 7.4 and for the akinetic-rigid subjects was 7.7, and it was thus even closer in magnitude than for the entire sample that included those with more severe PD.

## 4. Discussion

Medication response in a community-dwelling population of PD subjects was assessed by performing Motor UPDRS exams in the practically defined OFF as well as ON states. Subjects were examined after taking their usual prescribed dose of PD medications; thus, there was no standardization or specialist-directed optimization of medication regimens. This is representative of the treatment obtained in a community setting outside of tertiary care centers. We found that PD subjects with higher disease burden, evidenced by higher Motor UPDRS OFF scores, had a greater response to medications. The greater change may be in part a reflection of regression to the mean. Thus, PD symptoms clearly improved and to a greater degree when the baseline disease manifestations were more severe. There was no similar trend when symptom improvement was plotted against ON scores, suggesting that medications were titrated to a similar level of symptom improvement.

We did not identify a specific characteristic that allowed for prediction of better response to medications or higher differences between Motor UPDRS OFF and ON scores. Subdivision by medication classes used revealed similar medication response. There was a trend toward less response in the no levodopa group—which could support the knowledge that levodopa elicits greater symptom improvement than dopamine agonists or other medications [3, 4]—but this was not statistically significant. However, it must be noted that while we examined patients in the best defined OFF state, this only strictly applies to levodopa, as the other PD medications subjects were taking tend to have much longer half-lives. Even levodopa would likely need to be eliminated for days to weeks to completely wash out [7, 8].

Subjects on dopamine agonist without levodopa had lower medication OFF scores compared to the levodopa groups which could be explained by an incomplete wash-out of the dopamine agonist symptomatic benefit given the longer half-life of these agents. Alternatively, we may consider that subjects on dopamine agonists without levodopa may be in earlier stages of disease and thus have lower OFF scores. While disease staging by Hoehn and Yahr scores across the three medication subgroups did not meet statistical cutoffs for significance, a slightly higher percentage of the dopamine agonist without levodopa compared to the levodopa subjects were in Hoehn and Yahr stages 1 and 1.5. No dopamine agonist without levodopa subjects were in Hoehn and Yahr stages 4 or 5.

In addition, all PD subtypes responded similarly to medications regardless of baseline level of impairment. Akinetic-rigid PD subjects had a higher Motor UPDRS OFF score representing greater overall motor impairment and higher Hoehn and Yahr stage (Table 4), yet these symptoms responded to a similar degree as tremor-dominant and mixed subtypes. This is contrary to the report by Rajput et al., documenting a lower proportion of akinetic-rigid patients responding to levodopa [5]. Accounting for this difference, we consider that subjects in Rajput's et al., study were not assessed in the OFF and ON medication states as in our study. In addition, it must be considered that there may have been greater measurable improvement of more severe symptoms in the akinetic-rigid group or less measurable improvement of less severe symptoms in the tremor-dominant group.

The Motor UPDRS point improvement ranged from 6.1–8.5 among the various subgroups analyzed. We do not have a comparison in the literature for whether this magnitude of symptom control is similar to other community-based patient populations. However, Shulman and colleagues have reported that clinically important differences (CIDs, as defined as improvement notable to patients) in Motor UPDRS scores in their tertiary care referral center were 2.3–2.7 points (minimal CID), 4.5–6.7 points (moderate CID) and 10.7–10.8 points (large CID) [2]; analysis of the minimal CID within a clinical trial population of early PD patients participating in two dopamine agonist studies was estimated to be 5 points on the Motor UPDRS scale [9]. On the basis of these ranges, our subjects' level of improvement on their typical PD medication dosages falls within ranges that are likely clinically significant.

This study presents PD medication practices in a community dwelling population. Möller and colleagues reported on drug classes used in PD treatment in Germany from a general population sample revealed that 94.2% of patients were treated with levodopa with an average daily dose of 599 mg [10]. Sixteen percent of their PD population under the age of 70 were on levodopa alone without using dopamine agonists, and thus they reported that best treatment practices are not necessarily put into effect in the general population. They did not perform neurologic examinations to ascertain level of medication response.

Schrag and Quinn described treatment in a community-based population of PD patients specifically investigating the occurrence of dyskinesias and motor fluctuations, symptoms that emerge later in disease typically [11]. Again, there was no report of neurologic examination to document motor symptom severity.

Recently, Josephs et al., reported on a new PD subtype defined as benign tremulous Parkinsonism which, is defined as having tremor as the initial and most prominent symptom, having mild nontremor symptoms, very mild progression of disease over a long duration with absence of gait problems and disability except for tremor [12]. We are unable to determine whether any of our tremor-dominant subjects fit into this diagnostic criteria given that slow disease progression must be quantified for the diagnosis. Future assessments will be performed to determine if this subtype can be identified in our population to see if their treatment responses differ from other subtypes.

Our findings describing symptom responsiveness to PD medications in a general community setting reveal that there is clear improvement in symptom control irrespective of medication classes used or subtype of PD. The degree of symptom control is within a range determined to be clinically meaningful by previous studies. Future studies will investigate whether this degree of motor improvement correlates with health-related quality of life.

## Figures and Tables

**Table 1:** Sample demographics (*n* = 193).

Characteristic	Total *N* (%) or mean (SD)
Mean age, yrs, (SD)	72.4 (9.2)
Male, *n* (%)	114 (59.1)
White, *n* (%)	152 (78.8)
College education, *n* (%)	62 (32.1)
Married, *n* (%) (*n* = 190)	145 (76.3)
Employed full/part-time (%)	35 (18.1)
Mean duration of PD, yrs, (SD)	5.2 (2.3)
Mean Hoehn and Yahr stage, (SD)	2.7 (0.8)
Hoehn and Yahr stage, *n* (%)	
Stage 1: Unilateral disease	24 (12.8)
Stage 1.5: Unilateral plus axial involvement	10 (5.3)
Stage 2: Bilateral disease, without impairment of balance	72 (38.8)
Stage 2.5: Mild bilateral disease with recovery on pull test	41 (21.8)
Stage 3: Mild-to-moderate bilateral disease; some postural instability; physically independent.	26 (13.8)
Stage 4: Severe disability; still able to walk or stand unassisted	9 (4.8)
Stage 5: Wheelchair bound or bedridden unless aided	5 (2.7)
Medication status, *n* (%)*	
Levodopa only	67 (34.9)
Levodopa and any other medication	108 (56.3)
Dopamine agonists without levodopa	17 (8.9)
PD subtype, *n* (%)	
Tremor-dominant	33 (17.1)
Akinetic rigid	142 (73.6)
Mixed	18 (9.3)

* One subject excluded because they were on MAO-B inhibitors alone.

**Table 2:** Medications (*n* = 193).

Medication status	*N* (%)	Daily dosage (mg) mean (SD)
Levodopa only	67 (34.9)	
Carbidopa/levodopa	43 (22.3)	513.9 (254.6)
Carbidopa/levodopa (Sinemet CR or XR)	18 (9.3)	500.0 (205.8)
Levodopa and any other medication	108 (56.3)	
Pramipexole	46 (23.8)	2.3 (1.7)
Ropinirole	33 (17.1)	7.3 (6.9)
Bromocryptine	2 (1.0)	2.5 (0.0)
Carbidopa/levodopa	80 (41.5)	557.6 (307.2)
Carbidopa/levodopa (Sinemet CR or XR)	37 (19.2)	585.1 (296.9)
Amantadine	11 (5.7)	227.3 (46.7)
Trihexyphenidyl	4 (2.1)	5.5 (3.4)
Entacapone	23 (11.9)	747.8 (219.2)
Dopamine agonists without levodopa	17 (8.9)	
Pramipexole	10 (5.2)	2.4 (1.4)
Ropinirole	7 (3.6)	8.6 (4.7)

**Table 3:** Total Motor UPDRS ON and OFF medication by medication status (*n* = 193).

	Medication status*			
	Levodopa only (*n* = 67) mean (SD)	Levodopa and any other medication (*n* = 108) mean (SD)	Dopamine agonists without levodopa (*n* = 17) mean (SD)	ANOVA *P* value

Total Motor UPDRS ON medication	17.3 (11.9)^a^	17.3 (10.4)^a^	14.8 (8.1)^a^	0.65
Total Motor UPDRS OFF medication	24.6 (12.1)^a^	25.8 (12.0)^a^	20.9 (7.5)^b^	0.26
Difference between ON and OFF	7.3 (4.6)^a^	8.5 (5.4)^a^	6.1 (4.6)^a^	0.11

Hoehn and Yahr–OFF medication	*N* (%)	*N* (%)	*N* (%)	Chi-square *P*-value

1	4 (5.9)	4 (3.7)	2 (11.7)	0.76
1.5	3 (4.5)	8 (4.7)	1 (5.9)	
2	23 (34.3)	35 (32.4)	6 (32.3)	
2.5	22 (32.8)	27 (25.0)	5 (29.4)	
3	10 (14.9)	20 (18.5)	3 (17.7)	
4	2 (3.0)	10 (9.3)	0 (0.0)	
5	3 (4.5)	4 (3.7)	0 (0.0)	

**Table 4:** Total Motor UPDRS ON and OFF medication by PD subtype (*n* = 193).

	PD subtype			
	Tremor-dominant (*n* = 33) mean (SD)	Akinetic rigid (*n* = 142) mean (SD)	Mixed (*n* = 18) mean (SD)	ANOVA *P* value

Total Motor UPDRS ON medication	12.1 (7.8)^a^	18.9 (11.4)^b^	13.2 (5.1)^a^	0.001
Total fMotor UPDRS OFF medication	19.3 (9.9)^a^	26.9 (12.1)^b^	20.9 (5.7)^a^	0.001
Difference between ON and OFF	7.2 (5.1)^a^	8.0 (5.4)^a^	7.7 (1.9)^a^	0.73
Levodopa dosage per day (mg)	551.6 (306.2)^a^ (*n* = 32)	613.6 (285.4)^a^ (*n* = 116)	569.6 (357.6)^a^ (*n* = 14)	0.54

Hoehn and Yahr–OFF medication	*N* (%)	*N* (%)	*N* (%)	Chi-square *P*-value

1	5 (15.2)	5 (3.5)	0 (0.0)	0.003
1.5	5 (15.2)	4 (2.8)	3 (16.7)	
2	14 (42.4)	42 (29.6)	8 (44.4)	
2.5	5 (15.2)	46 (32.4)	3 (16.7)	
3	3 (9.1)	27 (19.0)	4 (22.2)	
4	1 (3.0)	11 (7.8)	0 (0.0)	
5	0 (0.0)	7 (4.9)	0 (0.0)	

UPDRS = Unified Parkinson's Disease Rating Scale.

*One subject excluded, because they were on MAO-B inhibitors alone.

^a, b^Means within a row with different letters differ significantly (*P* ≤ 0.05; pairwise *t*-test).
